# Mood Disorders and Severity of Addiction in Alcohol-Dependent Patients Could Be Mediated by Sex Differences

**DOI:** 10.3389/fpsyt.2019.00343

**Published:** 2019-05-31

**Authors:** Raul F. Palma-Álvarez, Laia Rodríguez-Cintas, Alfonso C. Abad, Marta Sorribes, Elena Ros-Cucurull, María Robles-Martínez, Lara Grau-López, Lourdes Aguilar, Carlos Roncero

**Affiliations:** ^1^Addiction and Dual Diagnosis Unit, Department of Psychiatry, Vall d’Hebron University Hospital–Public Health Agency, Barcelona (ASPB), CIBERSAM, Barcelona, Spain; ^2^Department of Psychiatry and Legal Medicine, Universitat Autònomade Barcelona, Barcelona, Spain; ^3^Institutde Neuropsiquiatria i Addiccions, Hospital del Mar, Barcelona, Spain; ^4^Psychiatry Service, Salamanca University Health Care Complex, Institute of Biomedicine, University of Salamanca, Salamanca, Spain

**Keywords:** alcohol, dependence, depression, suicide attempts, severity, sex differences, gender

## Abstract

**Background:** Alcohol dependence is highly prevalent in the general population; some differences in alcohol use and dependence between women and men have been described, including outcomes and ranging from biological to social variables. This study aims to compare the severity of alcohol dependence with clinical and psychopathological characteristics between sexes.

**Methods:** A cross-sectional descriptive study was conducted in alcohol-dependent outpatients; the recruitment period was 7 years. The assessment of these patients was carried out by obtaining sociodemographic characteristics and using the Semi-structured Clinical Interview for Axis I and II (SCID-I and SCID-II), European version of the Addiction Severity Index (EuropASI), Beck Depression Inventory (BDI), and State–Trait Anxiety Inventory (STAI) scales. Variables were compared and analyzed.

**Results:** The sample was composed of 178 patients (74.2% males and 25.8% females) with a mean age of 46.52 ± 9.86. No sociodemographic differences were found between men and women. Females had a higher rate of suicide attempts and depression symptoms at the treatment onset. When results of EuropASI were compared, females had worse psychological and employment results than males. According to consumption variables, males had an earlier onset of alcohol use, had more regular alcohol use, and develop alcohol dependence earlier than females.

**Conclusions:** According to results, there are sex-dependent differences (severity and other variables such as mood or suicide) in alcohol dependence. Thus, this may implicate the need of future specific research and treatment programs based on the specific necessities of each sex.

## Introduction

Alcohol dependence is a worldwide problem highly prevalent and associated with high morbidity and mortality (1–3). A total of 63.5 million people around the world had alcohol dependence in 2015 (3), and globally, the age-standardized rate for alcohol dependence is 843.2 per 100,000 people (3). For Western Europe, alcohol dependence seems to be higher, the rate being approximately 880.7 per 100,000 people (3). In Spain, 5% of the population aged between 15 and 64 years have a risk consumption of alcohol (4). It is important to highlight that the prevalence of high-risk drinking is higher in men than in women (5), and in Europe, this sex difference is also presented, reporting that 5.4% of men are alcohol-dependent as opposed to 1.5% of women (6). Additionally, alcohol consumption among women is increasing and sex differences are becoming increasingly close, especially in the younger groups (7–10).

Sex could be a modulating factor for several issues in mental and addictive disorders (11–13). Specifically in alcohol dependence, men have early onset of alcohol use, have higher craving levels, and consume more alcohol than women (5, 14, 15), but in contrast, women have a lower tolerance even with an equal alcohol blood level (16). Furthermore, women have more psychiatric comorbidity associated with alcohol consumption (17, 18), more medical and neurological problems related to consumption and dependence (7, 12, 19), and higher mortality rates (2, 19, 20). Although women start consuming the substance later, they become dependent faster than men (7). In the light of the foregoing, this is related to the *telescope effect*, which refers to a faster course of the addictive disorder among women (7, 21–23). It has been postulated that biological differences could support this hypothesis (9, 13, 17, 23). The telescope effect has also been described in alcohol-dependent patients with polydrug abuse (15, 24).

Deepening more on alcohol consumption and mental health issues, it has been described that alcohol-dependent women have more psychiatric comorbidity and tend to present a higher prevalence of anxiety and depressive symptoms and other mood disorders (18, 25–28). Depression may be an important factor during the approaching of alcohol use disorder because it could be a predictor factor related to treatment outcome, as it may be associated with craving and alcohol relapse (29–31). Also, lower baseline level in depression is associated with better treatment compliance (32) and better therapy response (33). Besides, depression is frequently associated with suicidal behavior in patients with substance use disorders (34). A recent meta-analysis on more than 400,000 participants reported a strong association between alcohol use disorder and suicidal behavior (35), and if it is analyzed as sex differences, women with alcohol use disorder have more suicidal attempts than men (36).

Finally, it has been described that there are sex differences in the difficulty of access to the clinical resources specialized in addictions between men and women (27, 37). These differences are due to several factors, such as social stigma, feelings of shame, denial, fear of losing their children, lack of specialized resources, and lower purchasing power (38, 39) These conditions could have overestimated the higher prevalence among men and would mean that the groups of women studied were more serious because of the fact that they seek treatment in a more advanced stage of the disorder. On the other hand, it is affirmed that women utilize and adhere to specialized resources in alcohol addiction and they achieve better outcomes overall (14, 36, 40). They also react positively to the consumption information (16). Thus, it would be helpful to thoroughly analyze the clinical differences due to the presence of mixed results in previous studies and the progressive changes observed in consumption patterns by sex. Furthermore, studies on this issue have been scarcely developed in Spain populations, especially if it is taking into account that not all studies have been performed with large samples and with validated scales and measures.

This study aims to compare the clinical and psychopathological features and the addiction severity according to sex in Spanish alcohol-dependent patients. It is hypothesized that sex differences in severity and in clinical and sociodemographic features will be found.

## Methods

This descriptive study was developed by the Addiction and Dual Diagnosis Unit of the Department of Psychiatry of Vall d’Hebron University Hospital of Barcelona between March 2007 and July 2014. The study was approved by Ethical Committee of Vall d’Hebron University Hospital according to the Declaration of Helsinki.

### Participants

Those subjects who were seeking for a new substance use disorder treatment at the Addiction and Dual Diagnosis Unit were invited to participate. The inclusion criteria were as follows: being over 18 years old, being in outpatient treatment for alcohol dependence, and having signed the informed consent form that was approved by the ethics committee of the hospital. The exclusion criteria were as follows: having a current dependence to other substances (excluding nicotine) and inability of the patient to participate in the evaluation process (because of intoxication, current serious psychiatric or somatic problems, and cognitive impairment or language barrier) at the time of the interviews. Patients did not receive financial compensation for their participation.

### Procedures

The patients had one psychiatrist visit and four psychological evaluation visits carried out by trained psychologists. Sociodemographic data, clinical information, and addiction variables were collected during the psychiatrist’s visit, while data related to addiction severity, presence of axis I and axis II mental disorders, depressive symptoms, anxiety symptoms, and suicidal behavior were collected during the psychologists’ visits.

### Evaluation Instruments

- *Sociodemographic and clinical questionnaire:* An *ad hoc* questionnaire that assesses sociodemographic variables, including age, marital status, education, employment, clinical relevant variables.
- *European version of the Addiction Severity Index (EuropASI):* Semi-structured interview, which measures the severity of addiction in different areas: medical, employment, alcohol consumption, use of other drugs, legal problems, family and social relationships, and psychological state. Scores range from 0 to 1; higher scores indicate higher severity (41).*Semi-structured Clinical Interview for Axis I and II (SCID-I and SCID-II):* These interviews were performed for evolution of Axis I and II disorders from the *Diagnostic and Statistical Manual of Mental Disorders fourth edition (DSM-IV)* (42, 43).*Beck Depression Inventory (BDI):* Self-administered questionnaire that measures the severity of current depressive symptoms (44).
- *State–Trait Anxiety Inventory (STAI):* Self-administered questionnaire that measures the current anxiety symptoms of the patient (45).

### Data Analysis

A descriptive analysis was conducted. In order to compare mean values between men and women, Student’s *t* test for quantitative variables and chi-square test for categorical variables were used. Adjustment of the results by Bonferroni correction was performed. A logistic regression analysis with forward selection was conducted using sex as dependent variable. Statistically significant variables obtained after bivariate analysis were chosen as independent variables. The statistical program used was the SPSS version 20.

## Results

**Figure 1:**
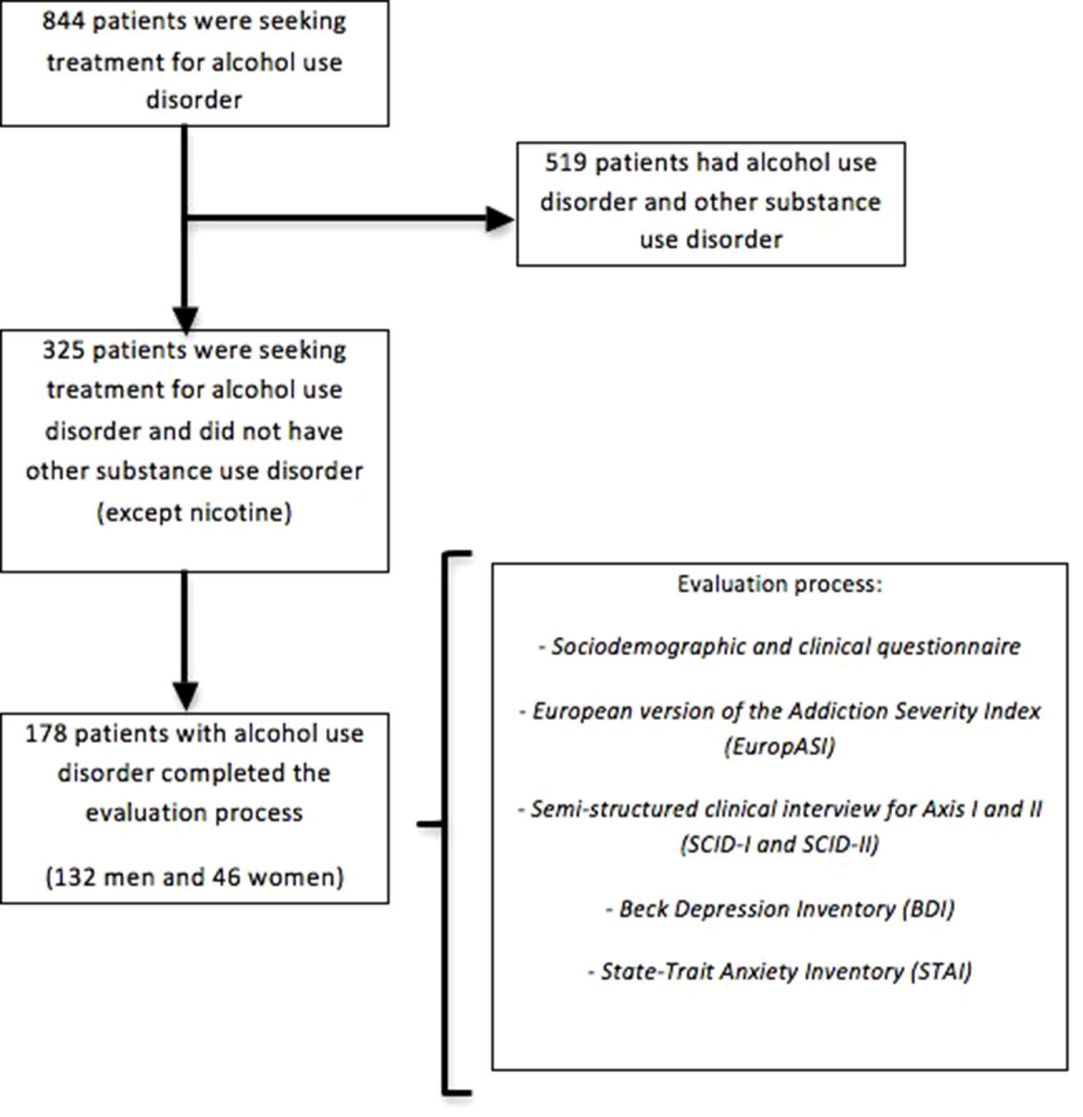
Sample flowchart.

From an initial sample of 844 patients under alcohol dependence treatment, the subgroup of patients with other substance dependence (except nicotine) was excluded in order to obtain a more homogeneous sample. A total of 325 patients were selected, and from this group, only 178 patients completed the evaluation process (74.2% men vs. 25.8% women; see **Figure 1**). The mean age at the first contact in our unit was 46.52 ± 9.86. Previously, we ensured that there were no significant differences between sexes regarding the antecedent of previous treatments in other clinical centers. In our sample, women seek treatment at an age equal to or greater than men.

It is observed that women present more depressive symptoms (measured with BDI) at the beginning of the treatment. No differences were found on nicotine use. Regarding the other sociodemographic and clinical variables, no differences were found between men and women (**Table 1**).

**Table 1:** Demographic, clinical, and psychiatric variables and diagnoses of the sample.

	DEMOGRAPHIC VARIABLES				
	*N* = 178	Men (*n* = 132)	Women (*n* = 46)	*t*	*p*
	Mean ± SD	Mean ± SD	Mean ± SD		
**AGE**	46.52 ± 9.86	45.84 ± 9.73	48.48 ± 10.09	1.57	0.119
**CIVIL STATUS**	%	%	%	χ^2^	*p*
Single	26.19	29.13	17.07	4.98	0.173
Married/partnered	34.52	33.07	39.02		
Separated/divorced	37.5	37	39.02		
Widow	1.78	0.79	4.88		
**LIVING WITH**	%	%	%	χ^2^	*p*
Partner and children	14.81	14.52	15.79	12.69	0.123
Partner	14.81	11.29	26.31		
Children	7.41	3.23	21.05		
Parents	14.81	24.2	10.53		
Family	9.88	9.68	10.53		
Friends	1.23	1.61	–		
Alone	22.22	24.19	15.79		
Protected environment	6.17	8.06	–		
Unstable	2.47	3.23	–		
**ACADEMIC LEVEL**	%	%	%	χ^2^	*p*
Primary (or lower)	43.6	45.9	36.6	1.083	0.364
Secondary (or upper)	56.4	54.1	63.4		
**EMPLOYMENT**	%	%	%	χ^2^	*p*
Active	27.22	30.71	16.67	3.141	0.076
	**CLINICAL VARIABLES**				
	%	%	%	χ^2^	*p*
**Psychiatric history**	51.92	52.5	50	0.046	0.830
Nicotine use	76.3	79.2	67.7	1.593	0.222
Psychotic symptoms	18.5	22.7	7.3	**4.696**	**0.034**
Suicide attempts	19.1	12.7	34.6	**5.721**	**0.035**
	%	%	%	χ^2^	*p*
**Medical history**	59.4	59.2	60	1.254	0.534
Infectious Dis.	6.3	7	4.4	1.656	0.647
Cardiological Dis.	12.6	15.5	4.4	4.55	0.208
Hepatic Dis.	21.4	20.2	25	2.477	0.479
**BDI**	16.4	14.65	20.54	**2.844**	0.006*
**STAI-E**	58.19	58.11	58.47	0.059	0.953
**SCID I (111/39)**	%	%	%	χ^2^	*p*
Mood Disorder	30.7	26.4	42.5	3.592	0.072
Anxiety Disorder	17.1	14.3	25	2.386	0.144
**SCID II**	%	%	%	χ^2^	*p*
Personality Disorder	19.2	20.7	15	0.620	0.492

*Significant with Bonferroni.

All the significant results are in bold.

By comparing the variables related to the characteristics of consumption, men start drinking earlier, consume alcohol regularly, and develop dependence earlier than women. When the amount is observed, men consume more Standard Drinks per day (1 SD = 10 g of alcohol in Spain) in the past 6 months (**Table 2**).

**Table 2:** Alcohol consumption chronologic features.

	*N = 178*		*Men (n = 132)*		*Women (n = 46)*		*t*	*p*
	Mean	SD	Mean	SD	Mean	SD		
**Alcohol consumption in the last 6 months, Standard Drinks (SD)/day**	13.14	13.30	14.5	14	8.83	9.84	**2.61**	0.011*
**Alcohol last month, SD/day**	8.90	10.53	9.87	11.27	6.18	7.57	**2.25**	**0.027**
**Age of first contact**	16.06	5.42	15.37	4.294	18.12	7.55	**2.94**	0.004*
**Age at onset of regular consumption**	24.97	10.36	22.57	8.543	31.95	12.02	**4.73**	0.000*
**Years of regular consumption**	19.66	13.8	20.96	13.19	16.25	14.98	1.602	0.112
**Years from the first contact to the onset of dependence**	8.87	8.6	7.44	7.66	13.05	9.89	**3.384**	0.000*
	%		%		%		χ^2^	*p*
**Abuse of other substance (*DSM-IV*)**	19.5		22.5		11.6		2.345	0.174								

*Significant with Bonferroni.

All the significant results are in bold.

It is observed that alcohol-dependent women had more problems in the areas related to employment status and psychological state, which were measured by the EuropASI score (**Table 3**). However, alcohol-dependent men showed more problems in the area of other drug consumption (throughout life).

**Table 3:** EuropASI subareas in the evaluation of the severity of addiction.

	*N* = 178		Men (*n* = 132)		Women (*n* = 46)		*t*	*p*
	Mean	SD	Mean	SD	Mean	SD		
**Medical**	0.31	0.35	0.29	0.35	0.39	0.37	1.74	0.084
**Employment**	0.53	0.33	0.49	0.33	0.63	0.3	**2.46**	0.015*
**Alcohol**	0.37	0.31	0.37	0.31	0.36	0.31	0.31	0.761
**Drugs**	0.09	0.23	0.1	0.25	0.04	0.11	**2.3**	0.023*
**Legal**	0.06	0.17	0.07	0.17	0.04	0.15	0.78	0.437
**Familiar/Social**	0.29	0.28	0.27	0.28	0.34	0.28	1.41	0.159
**Psychological**	0.33	0.23	0.3	0.23	0.4	0.21	**2.47**	0.015*

*Significant with Bonferroni.

All the significant results are in bold.

Finally, multivariate analysis is depicted in **Table 4**. There were statistically significant sex-dependent differences associated with suicide attempts, EuropASI (*Psychological* and *Employment* areas), age at onset of regular consumption, and BDI.

**Table 4:** Logistic regression analysis.

	Wald	p	OR	IC 95%
**Suicide attempts**	4.903	**0.027**	15.500	**1.370–17.538**
**BDI**	8.779	**0.003**	1.055	**1.018–1.093**
**Age at onset of regular consumption**	9.568	**0.002**	1.077	**1.027–1.128**
**Employment**	4.823	**0.028**	3.458	**1.143–10.463**
**Psychological**	5.109	**0.024**	5.941	**1.267–27.855**

All the significant results are in bold.

## Discussion

The obtained results show the existence of sex-related differences in alcohol consumption in the Spanish population. Alcohol-dependent women who sought treatment in our center presented a later drinking onset and consumed less amounts of alcohol than men. However, they tended to seek treatment in our unit later than men and had more depressive symptoms. These results are in line with previous research in other countries. In many recent studies, it has been described that there is an increasing prevalence of alcohol dependence among women (8–10), and they have a more severe alcohol use disorder (2, 11, 12, 19).

Regarding clinical variables, sex differences are observed. It was found that women who sought treatment for alcohol dependence presented more depressive symptoms at the beginning of the treatment, which had been previously described in other countries (26, 29, 46, 47). It has been described that depressive symptoms in alcohol-dependent women are related to a greater craving (which can lead to a relapse), and this issue has not been observed in the male sex (30, 31, 47, 48). Therefore, depression becomes an important and critical issue during the approaching for alcohol use disorder, not only for its prevalence but also because depression levels are associated with some treatment outcomes (32, 33). Furthermore, depression and suicidal behavior are closely related; in the current sample, suicide behavior was significant at multivariate analysis. This is in line with some authors that have described that women with alcohol dependence are at higher risk for unplanned or planned attempts (49). Therefore, alcohol dependence is an important risk factor for suicidal behavior, and this concern should always be evaluated for an adequate and integral management (35, 36). Differences in relation to the presence of psychotic symptoms between men and women are suggested, which would be consistent with previous studies that evaluate both the presence of alcohol-induced psychotic symptoms and delirium tremens (50, 51), but these results are nonsignificant when Bonferroni correction was performed.

Although men consumed, in general, larger amounts of alcohol and began consumption earlier than women, taking into account the results of the EuropASI scale, it could be suggested that alcohol dependence was more serious in women because they had higher severity in two of the seven areas measured by the EuropASI scale (*Employment* and *Psychological* areas) and less severity in one of the seven areas (*Drugs* area), showing no differences in the other areas. It is also observed that the employment area in EuropASI was more affected in women. The improvement of economic and work conditions in women could be associated with an increased consumption of alcohol in women. Recent studies have found that changes in economic and work conditions could be contributing to the convergence in sex differences (8). Scores in the *Psychological* area were worse in women; this could be related to the depressive symptoms in this group. This finding is similar to other research that described that the *Psychological* area in EuropASI is worse in women in several settings, such as outpatient centers and therapeutic communities (22, 52). Thus, analyzing all the results, in the current sample, there seems to exist a certain degree of *telescope effect*, in line with other research in similar samples in other countries (7, 21, 23).

This study should be analyzed regarding its limitations. Thus, the transversal nature and the incomplete representation of all the alcohol-dependent patients become an important issue (participants who do not seek treatment or patients with mixed dependences). Furthermore, we did not analyze differences regarding nicotine use, as this substance could be related to psychopathology and sex differences. It is also possible that the men who seek treatment tend to minimize or deny their depressive symptoms. Finally, the recruitment period (2007–2014) could generate outdated information; however, these data could contribute to understand this issue in Spanish populations with alcohol use disorder and allow to compare future research on this issue. On the other hand, the study was conducted in a large homogeneous sample of patients who are only alcohol-dependent in an outpatient treatment center. Consequently, the results are representative of the clinical activity in a real clinical setting.

The current results provide new information that has been scarcely explored in large samples in Spanish populations with alcohol use disorder. The increased presence of depressive symptoms and the greater severity of addiction make women a particularly vulnerable group. Thus, as other authors recommended, it could be suggested that it is necessary to consider the special clinical characteristics of women when planning the treatments (7, 53, 54). Furthermore, regarding the results of this study, it seems to be advisable that the implementations of programs to detect depressive symptoms and to prevent suicide, especially in alcohol-dependent women, are needed.

## Ethics Statement

The research was approved by Ethical Committee of Vall d’Hebron University Hospital according to Declaration of Helsinki.

## Author Contributions

CR and LG-L designed the study. RP-A, LR-C, AA, MR-M, and ER-C collected the sample. LR-C, MS, and MR-M performed psychological evaluation. RP-A, LR-C, LG-L, and LA conducted the analyses and prepared the data. AA, MS, MR-M, ER-C, and LA performed the literature review. RP-A, AA, ER-C, and LA wrote the initial version of this manuscript. All authors edited, read, and approved the last version of this manuscript.

## Funding

This research did not receive any specific grant from funding agencies in the public, commercial, or not-for-profit sectors.

## Conflict of Interest Statement:

The authors declare that the research was conducted in the absence of any commercial or financial relationships that could be construed as a potential conflict of interest.
